# Pharmacotherapy for the Treatment of Overweight and Obesity in Children, Adolescents, and Young Adults in a Large Health System in the US

**DOI:** 10.3389/fendo.2020.00290

**Published:** 2020-05-13

**Authors:** Kathryn S. Czepiel, Numa P. Perez, Karen J. Campoverde Reyes, Shreya Sabharwal, Fatima Cody Stanford

**Affiliations:** ^1^Department of Pediatrics, Massachusetts General Hospital and Harvard Medical School, Boston, MA, United States; ^2^Department of General Surgery, Massachusetts General Hospital and Harvard Medical School, Boston, MA, United States; ^3^Neuroendocrine Unit, Massachusetts General Hospital and Harvard Medical School, Boston, MA, United States; ^4^Liver Research Center, Beth Israel Deaconess Medical Center, Boston, MA, United States; ^5^Department of Bioethics, Harvard Medical School, Boston, MA, United States; ^6^Pediatric Endocrinology, Massachusetts General Hospital and Harvard Medical School, Boston, MA, United States; ^7^MGH Weight Center, Massachusetts General Hospital, Boston, MA, United States

**Keywords:** children, adolescents, young adults, overweight, obesity, pharmacotherapy, weight loss medications, anti-obesity medications

## Abstract

Lifestyle modifications focused on diet, physical activity, and behavior have a modest impact on weight reduction in children, adolescents, and young adults (YA) with overweight and obesity. Several anti-obesity medications (AOMs) have been approved by the Food and Drug Administration (FDA) for use among adult patients with a body mass index (BMI) ≥27 kg/m^2^ and at least one obesity-related illness. However, only two FDA-approved AOMs are available for use in children and adolescents, which leads to the frequent off-label use of adult AOMs among this population. We sought to investigate current prescribing patterns of AOMs from school age through to young adulthood in a large unified health system. Using a centralized clinical data registry containing the health data of ~6.5 million patients, individuals aged 5–25 years old with overweight and obesity who were taking one of eight commonly prescribed AOMs from 2009 to 2018 were extracted. A total of 1,720 patients were identified, representing 2,210 medication prescribing instances. The cohort was further stratified as children (5–12 years old), adolescents (13–18 years old), and YA (19–25 years old). The mean BMI at the time of medication initiation was 34.0, 39.1, and 39.6 kg/m^2^, respectively, which corresponded to a BMI *z*-score (BMIz) of 2.4 and 2.3 for children and adolescents, respectively. Metformin was the most commonly prescribed medication across all ages, including off-label use for weight-loss among children and adolescents. The most commonly off-label prescribed AOM among YA was topiramate. Multivariable analyses demonstrated phentermine was the most effective AOM, with a 1.54% total body weight among YA (*p* = 0.05) and a 0.12 decrease in BMIz among adolescents (*p* = 0.003) greater final weight loss when compared to the respective overall frequency-weighted means. Our study demonstrates a statistically significant weight loss among adolescents and young adults on select pharmacotherapy. The small magnitude of this effect should be interpreted carefully, as it is likely an underestimate in the absence of a true control group. Pharmacotherapy should therefore be considered in conjunction with other multimodal therapies such as lifestyle modification and metabolic and bariatric surgery when treating overweight and obesity.

## Introduction

The most recent analysis of obesity prevalence using the National Health and Nutrition Examination Survey (NHANES) database shows that 1 in 5 children in the United States have obesity (1). Children in high-risk groups such as those with a genetic predisposition to obesity (2), and those with poor quality early lifestyle and dietary behaviors (3) are likely to develop childhood obesity which often propagates into adulthood along with obesity-related illnesses (1, 4–9).

Current treatment modalities for childhood obesity are multidisciplinary in nature with a significant preference toward lifestyle modifications that target dietary and behavioral change as the foundation for treatment in most children, adolescents and young adults who present for care. However, a 2017 Cochrane Review found low quality evidence that lifestyle modification focused on diet, physical activity, and other behaviors reduces BMI in adolescents (10). According to this review, the mean change in BMI was −1.18 kg/m^2^ with a weight loss (WL) of 3.67 kg (95% CI −5.21 to −2.13) across 28 randomized-clinical trials, encompassing 2,774 participants ages 12–17.

The rise of obesity over the past 30 years, especially with high rates of severe obesity in children living in non-metropolitan areas (11), calls for more aggressive combination therapies that include behavioral modification, medications, and surgical interventions (12, 13). According to the most recently available data from the NHANES, the prevalence of severe obesity from 2015 to 2016 was 1.9% among children and adolescents 2 to 19 years old, with 4.5% of adolescents age 16–19 years old affected by severe obesity (1). While strides have been made toward encouraging the utilization of metabolic and bariatric surgery (MBS) for pediatric patients with severe obesity, with definitive guidance from organizations such as the American Academy of Pediatrics (AAP) and the American Society of Metabolic and Bariatric Surgery (ASMBS) (14–17), there remains a gap in care for patients who have been refractory to lifestyle modifications but do not meet criteria for MBS.

The current use of pharmacotherapy for the treatment of obesity in the pediatric population is limited (18). Compared to prescribing patterns for the treatment of other pediatric chronic diseases such as type 2 diabetes mellitus (T2DM), anti-obesity medications (AOMs) are disproportionately underutilized in relation to the disease burden (19), likely due to the lack of current guidelines addressing their use. Notably, the current Endocrine Society practice guidelines recommend that AOM use should be confined to clinical trials for pediatric patients with obesity (20). Furthermore, only two AOMs are currently approved by the Food and Drug Administration (FDA) for management of obesity in the pediatric population: orlistat for patients ≥12 years and phentermine in those >16 years (21, 22). Since most medications with the potential benefit of WL are used either for a separate indication or used off-label for the treatment of obesity, there is limited reliable data available on the utilization of these medications and weight outcomes (23). The purpose of this study was to ascertain current prescribing practices of these AOMs among the pediatric population in a large unified health system, and second, to assess the effect of these medications on weight status.

## Methods

### Data Source

The centralized clinical data registry of a large unified health care system, consisting of two large academic medical centers and three community teaching hospitals was used to identify the study cohort. This clinical data registry contains electronic health records (EHR) data spanning over 30 years for ~6.5 million patients, and allows for research cohort identification using a combination of user-defined characteristics such as patient demographics, diagnoses, medications, etc. Institutional Review Board (IRB) approval was obtained prior to extraction of any patient health data.

### Patient Population

Patients ages 5–25 years old with overweight and obesity, as indicated by *International Classification of Diseases* (ICD) 9 codes V85.21-25, V85.3, V85.4, V85.53-54, and *International Classification of Diseases* (ICD) 10 codes Z68.25-29, Z68.3, Z68.4, Z68.53-54, who were on one of several commonly prescribed AOMs between the years 2009 and 2018 were identified. The following fourteen FDA- and non-FDA-approved medications or association of medications were included in the initial extraction (listed in alphabetical order): bupropion, bupropion/naltrexone, canagliflozin, exenatide, liraglutide, lorcaserin, metformin, naltrexone, orlistat, phentermine, phentermine/topiramate, pramlintide, topiramate, and zonisamide.

### Data Extraction

Patient demographics, to include age at the time of medication initiation, sex, race, and primary insurance were extracted. The patient cohort was then stratified into three age categories: (1) Children: patients 5–12 years old; (2) Adolescents: patients 13–18 years old; and (3) Young adults: patients 19–25 years old. Clinical information to include medical and surgical history, medication prescriptions, and measured heights and weights were also obtained. Among children and adolescents, overweight was defined as a BMI 85th to 95th percentile for age and sex, and obesity was classified as class I (BMI 95th to 120% of 95th percentile for age and sex),class II obesity (BMI > 120–140% of the 95th percentile for age and sex, or 35 to <40 kg/m^2^, whichever is lower), and class III (BMI >140% of the 95th percentile for age and sex, or >40 kg/m^2^, whichever is lower) (14, 24, 25). For young adults, overweight was defined as BMI 25–30 kg/m^2^, and obesity was classified as class I (BMI 30 to <35 kg/m^2^), class II (BMI 35 to <40 kg/m^2^), and class III (BMI ≥ 40 kg/m^2^) (24–27). Additionally, BMI *z*-scores (BMIz) were calculated for the children and adolescent cohorts based on the CDC growth charts (28–30).

The following obesity-related illnesses were coded: hypertension (HTN), dyslipidemia (DL), type 2 diabetes mellitus (T2DM), obstructive sleep apnea (OSA), obesity-hypoventilation syndrome, non-alcoholic fatty liver disease (NAFLD), gastroesophageal reflux disease (GERD), idiopathic intracranial hypertension (IIH), depression, anxiety, and personality disorders (Supplemental Table 1).

The date of medication initiation was available as a structured data field directly from the clinical data registry. On the other hand, the date of medication discontinuation, though also available as a structured data field, was deemed unreliable due to known discrepancies between actual medication discontinuation and documentation in patients' EHR. Instead, the date of medication discontinuation was determined by direct examination of provider clinic notes, which ensured its accuracy. For patients who underwent MBS while on a study medication, the date of discontinuation was set as the date of surgery, and for patients who remained on a medication at the time of data extraction (Aug 27, 2019), the discontinuation date was set as the date of data extraction. Given the possibility that patients may have taken multiple AOMs simultaneously, the dates of initiation and discontinuation were recalculated to be exclusive of those of any other overlapping medications, which allowed for determination of the individual effects of each medication on a patient's weight. A medication instance was defined as the period during which a patient took a single medication exclusively without interruption. Hence, one patient could have had multiple instances of the same medication, as well as several instances of multiple medications.

Once the exclusive dates had been determined for each medication instance, all weights recorded for each patient were extracted and the following pertinent weights were defined: (1) Starting weight: the weight recorded closest to medication initiation (within 90 days pre and post); (2) Nadir weight: Lowest weight recorded during a medication instance; (3) End weight: the weight recorded closest to medication discontinuation (within 90 days pre and post). Maximum WL was defined as the starting weight minus the nadir weight. Final WL was defined as the patient's starting weight minus the end weight, hence a positive number indicates WL, while a negative number indicates weight gain. WL was represented as change in BMIz (i.e., ΔBMIz) for children and adolescents, and as percentage of total body weight (%TBW) for young adults. After the initial cohort had been identified, only medications with at least 5 medication instances with complete weight data (i.e., start, nadir, and end weights recorded in chart) were kept, which prompted the elimination of six medications: bupropion/naltrexone, canagliflozin, exenatide, lorcaserin, phentermine/topiramate, pramlintide. Table 1 contains the eight medications ultimately included in our study. Given that these medications are not all uniquely prescribed for weight-loss, we also flagged the following pertinent medical conditions as potential on-label indications for medication prescription: epilepsy, tobacco use/dependence, alcohol dependence/abuse, opioid dependence/abuse (Supplemental Table 1).

**Table 1:** Weight-loss medications commonly prescribed to children, adolescent, and young adults with obesity (31, 32).

**Generic name**	**Trade name(s)**	**Mechanism(s) of action in obesity treatment**	**FDA approved for weight loss in pediatrics (age)**	**Pediatric recommendation and strength of evidence**
Bupropion	Wellbutrin	Increases extracellular dopamine in the nucleus accumbens, an important part of the neural circuitry of reward	No	
Liraglutide	Victoza, Saxenda	Increases insulin release through activation of the GLP-1 receptor, which causes increased cAMP	No, approved for use to treat T2DM in children >10 yo	
Metformin	Glucophage	Improves insulin sensitivity through increased peripheral glucose uptake and utilization	No, approved for use to treat T2DM in children >10 yo	
Naltrexone	Revia	Not completely understood, but may modulate the opioid receptor to decrease food cravings	No, off label use for alcohol and/or opioid dependence	
Orlistat	Xenical, Alli	Block fat absorption through inhibitory binding of pancreatic and gastric lipase in the gastrointestinal tract	Yes (≥12 yo)	IIa^‡^, Category B^¥^ for long-term use
Phentermine	Adipex and others	Appetite suppression through hypothalamic release of catecholamines	Yes (>16 yo)	IIb^§^, Category B^¥^ for short-term use
Topiramate	Topamax	Appetite suppression through potential augmentation of GABA	No	
Zonisamide	Zonegran	Not completely understood, but facilitates dopaminergic and serotonergic neurotransmission	No	

T2DM, Type II Diabetes Mellitus.

‡ Strength of recommendation IIa indicates that the treatment is generally considered to be useful and is indicated in most cases (33).

¥ Strength of evidence Category B is based on data derived from meta-analyses of randomized controlled trials (RCT) with conflicting conclusions with regard to the directions and degrees of results between individual studies. RCTs involved small numbers of patients or had significant methodological flaws (33, 34).

§ *Strength of recommendation IIb indicates that the treatment may be useful, and is indicated in some, but not most cases (34)*.

### Statistical Analysis

Univariate statistics comparing patient demographics and medication details (i.e., medication duration, BMI and BMIz at time of medication initiation, nadir and WL, etc.) were obtained using Student's *t*-test and Pearson's chi_2_. Multivariate analyses to ascertain the effects of each medication on a patient's weight were also performed using linear regression clustered at the patient level and adjusted for patient age at the time of medication initiation, sex, race, primary insurance, obesity class at time of medication initiation, and number of obesity-related illnesses. Rather than arbitrarily selecting one of the medications as reference for analysis, the change in ΔBMIz or %TBW associated with each medication was compared to the overall frequency-weighted mean (mean).

Lastly, in order to ascertain the effects of these medications when prescribed specifically for WL, all patients with a potential on-label indication for prescription were excluded and all aforementioned unadjusted and adjusted analyses were repeated. The following specific groups were excluded: (1) Patients with diabetes on metformin or liraglutide; (2) Patients with epilepsy on topiramate or zonisamide; (3) Patients with depression, anxiety, or tobacco use/dependence disorder on buproprion; (4) Patients with opioid or alcohol dependence/abuse disorder on naltrexone.

Table cells with 10 patients or less were represented as such to preserve patient privacy. All statistical analyses were performed using the Stata statistical software package (version 15.1; StataCorp LP, College Station, TX, USA).

## Results

A total of 1,720 patients were identified, consisting of 83 children, 492 adolescents, and 1,145 young adults, which together represented 2,210 medication instances. A starting weight and BMI were available for roughly 75% of patients (Table 2). The mean BMI at the time of medication initiation among children, adolescents, and young adults was 34.0, 39.1, and 39.6kg/m^2^, respectively. This corresponded to a BMIz of 2.4 and 2.3 for children and adolescents respectively. Most patients (1,343; 78.1%) were prescribed a single medication during the study period. A higher proportion of young adults were female (82.3%) than among adolescents (70.9%) or children (54.2%). Most patients were white (59.1%) and privately insured (58.1%). A total of 268 patients underwent MBS during the study period, consisting of 181 (10.5%) sleeve gastrectomies, 87 (5.1%) laparoscopic Roux-en-Y gastric bypasses, and ≤ 10 laparoscopic adjustable gastric band placements. Information on obesity-related illnesses can be found in Table 2.

**Table 2:** Patient demographics and clinical characteristics.

	**Children**	**Adolescents**	**Young adults**	**Overall**
	***N* = 83**	***N* = 492**	***N* = 1,145**	***N* = 1,720**
Age at medication initiation, mean (95% CI)	10.7 (10.3–11.0)	15.8 (15.7–16.0)	22.6 (22.5–22.7)	20.1 (19.9–20.3)
Starting weight/BMI recorded in chart, n (%)	63 (75.9)	388 (78.9)	828 (72.3)	1,279 (74.4)
Starting BMI, mean kg/m^2^ (95% CI)	34.0 (32.0–35.9)	39.1 (38.2–39.9)	39.6 (39.0–40.2)	39.2 (38.7–39.7)
Starting BMI Z-score, mean (95% CI)	2.4 (2.3–2.5)	2.3 (2.3–2.4)	N/A	2.3 (2.3–2.4)
Obesity class, *n* (%)				
Overweight	≤ 10	36 (8.5)	125 (13.6)	162 (11.5)
Class I (mild)	23 (32.9)	108 (25.4)	208 (22.7)	339 (24.0)
Class II (moderate)	19 (27.1)	118 (27.8)	222 (24.2)	359 (25.4)
Class III (severe)	27 (38.6)	163 (38.4)	362 (39.5)	552 (39.1)
Number of weight-loss medications, *n* (%)				
1	68 (81.9)	351 (71.3)	924 (80.7)	1,343 (78.1)
2	12 (14.5)	100 (20.3)	177 (15.5)	289 (16.8)
3	3 (3.6)	27 (5.5)	37 (3.2)	67 (3.9)
≥4	(0.0)	14 (2.9)	7 (0.6)	21 (1.2)
Sex, *n* (%)				
Male	38 (45.8)	143 (29.1)	203 (17.7)	384 (22.3)
Female	45 (54.2)	349 (70.9)	942 (82.3)	1,336 (77.7)
Race, *n* (%)				
White	40 (52.6)	285 (59.3)	666 (59.5)	991 (59.1)
Black	≤ 10	44 (9.2)	131 (11.7)	185 (11.0)
Hispanic	19 (25.0)	82 (17.1)	177 (15.8)	278 (16.6)
Asian/Pacific Islander	≤ 10	15 (3.1)	16 (1.4)	32 (1.9)
Native American	0	≤ 10	≤ 10	≤ 10
Other	≤ 10	53 (11.0)	124 (11.1)	183 (10.9)
Insurance, *n* (%)				
Private Insurance	50 (60.2)	300 (61.0)	649 (56.7)	999 (58.1)
Medicaid	33 (39.8)	164 (33.3)	404 (35.3)	601 (34.9)
Self-pay	(0.0)	17 (3.5)	32 (2.8)	49 (2.9)
Metabolic and bariatric surgery, *n* (%)				
Sleeve gastrectomy	0	46 (9.4)	134 (11.7)	181 (10.5)
Laparoscopic roux-en-Y gastric bypass	0	19 (3.9)	68 (5.9)	87 (5.1)
Laparoscopic adjustable gastric band	0	0	≤ 10	≤ 10
Obesity-related illnesses, mean (95% CI)	2.7 (2.3–3.0)	3.2 (3.0–3.4)	3.2 (3.1–3.3)	3.2 (3.1–3.3)
Obesity-related illnesses, *n* (%)				
Anxiety	45 (54.2)	302 (61.4)	723 (63.3)	1,070 (62.3)
Depression	28 (33.7)	261 (53.1)	668 (58.4)	957 (55.7)
Gastroesophageal reflux disease	21 (25.3)	177 (36.0)	490 (42.9)	688 (40.1)
Hypertension	16 (19.3)	111 (22.6)	369 (32.3)	496 (28.9)
Dyslipidemia	20 (24.1)	150 (30.5)	317 (27.7)	487 (28.4)
Type 2 diabetes mellitus	19 (22.9)	119 (24.2)	295 (25.8)	433 (25.2)
Attention-deficit hyperactive disorder	34 (41.0)	136 (27.6)	226 (19.8)	396 (23.1)
Obstructive sleep apnea	19 (22.9)	134 (27.2)	245 (21.4)	398 (23.2)
Nonalcoholic fatty liver disease	14 (16.9)	127 (25.8)	247 (21.6)	388 (22.6)
Personality Disorders	≤ 10	27 (5.5)	66 (5.8)	93 (5.4)
Idiopathic intracranial hypertension	≤ 10	15 (3.1)	42 (3.7)	57 (3.3)
Obesity hypoventilation syndrome	≤ 10	≤ 10	13 (1.1)	13 (0.8)
Other on-label indications, *n* (%)				
Epilepsy	13 (15.7)	81 (16.5)	147 (12.9)	241 (14.0)
Tobacco use/dependence	≤ 10	37 (7.5)	195 (17.1)	232 (13.5)
Alcohol dependence/abuse	0	14 (2.9)	117 (10.2)	131 (7.6)
Opioid dependence/abuse	0	≤ 10	68 (6.0)	68 (4.0)

*BMI, Body Mass Index*.

### Children

Overall, metformin was the most commonly prescribed medication among children (41; 49.4%). After excluding patients with potential on-label indications, metformin remained the most commonly prescribed medication, along with topiramate both at 52.2% (two patients were on both medications; Table 3). Most children continued to take the study medication at the time of data extraction (58.5%), while 22.0% had discontinued it for unknown/unspecified reasons. See Table 4 for the other common reasons for medication discontinuation.

**Table 3:** Weight loss medications—on and off-label use.

	**Young adults**	**Adolescents**	**Children**	**Overall**
	***N* = 1,145**	***N* = 492**	***N* = 83**	***N* = 1,720**
**ALL PATIENTS**				
Metformin	484 (42.3)	280 (56.9)	41 (49.4)	805 (46.8)
Topiramate	377 (32.9)	156 (31.7)	32 (38.6)	565 (32.8)
Bupropion	370 (32.3)	123 (25.0)	16 (19.3)	509 (29.6)
Phentermine	141 (12.3)	47 (9.6)	—	188 (10.9)
Zonisamide	32 (2.8)	15 (3.0)	—	47 (2.7)
Naltrexone	39 (3.4)	—	—	39 (2.3)
Orlistat	23 (2.0)	—	—	23 (1.3)
Liraglutide	23 (2.0)	—	—	23 (1.3)
**OFF-LABEL USE ONLY**				
	***N*** **=** **644**	***N*** **=** **299**	***N*** **=** **46**	***N*** **=** **989**
Metformin	255 (39.6)	173 (57.9)	24 (52.2)	452 (45.7)
Topiramate	298 (46.3)	122 (40.8)	24 (52.2)	444 (44.9)
Bupropion	21 (3.3)	10 (3.3)	—	31 (3.1)
Phentermine	141 (21.9)	47 (15.7)	—	188 (19.0)
Zonisamide	16 (2.5)	—	—	16 (1.6)
Naltrexone	18 (2.8)	—	—	18 (1.8)
Orlistat	23 (3.6)	—	—	23 (2.3)

**Table 4:** Reasons for medication discontinuation as recorded in the chart.

**Medication**	**Still taking, *n* (%)**	**Unknown reason, *n* (%)**	**GI symptoms, *n* (%)**	**No longer necessary, *n* (%)**	**Non-compliance, *n* (%)**	**No effect, *n* (%)**
**Young adults (19–25 years old)**						
Metformin	195 (44.7)	135 (31.0)	36 (8.3)	21 (4.8)	15 (3.4)	14 (3.2)
Topiramate	114 (35.6)	116 (36.3)	2 (0.6)	28 (8.8)	7 (2.2)	15 (4.7)
Bupropion	94 (30.6)	141 (45.9)	0 (0)	24 (7.8)	12 (3.9)	11 (3.58)
Phentermine	40 (34.8)	33 (28.7)	2 (1.7)	14 (12.2)	3 (2.6)	4 (3.5)
Zonisamide	8 (33.3)	11 (45.8)	0 (0)	2 (8.3)	0 (0)	1 (4.2)
Naltrexone	5 (19.2)	16 (61.5)	0 (0)	4 (15.4)	0 (0)	0 (0)
Orlistat	0 (0)	13 (72.2)	0 (0)	2 (11.1)	0 (0)	0 (0)
Liraglutide	9 (52.9)	6 (35.3)	1 (5.9)	1 (5.9)	0 (0)	0 (0)
Overall	465 (36.8)	471 (37.3)	41 (3.3)	96 (7.6)	37 (2.9)	45 (3.6)
**Adolescents (13–18 years old)**						
Metformin	137 (54.2)	63 (24.9)	11 (4.4)	9 (3.6)	17 (6.7)	4 (1.6)
Topiramate	57 (41.3)	47 (34.1)	1 (0.7)	7 (5.1)	6 (4.4)	5 (3.6)
Bupropion	38 (33.9)	46 (41.1)	0 (0)	10 (8.9)	2 (1.8)	2 (1.8)
Phentermine	18 (40)	16 (35.6)	1 (2.2)	0 (0)	1 (2.2)	0 (0)
Zonisamide	4 (40)	5 (50)	0 (0)	1 (10)	0 (0)	0 (0)
Overall	254 (45.5)	177 (31.7)	13 (2.3)	27 (4.8)	26 (4.7)	11 (2.0)
**Children (5–12 years old)**						
Metformin	27 (69.2)	7 (18.0)	1 (2.6)	2 (5.1)	2 (5.1)	0 (0)
Topiramate	15 (53.6)	6 (21.4)	0 (0)	3 (10.7)	0 (0)	3 (10.7)
Bupropion	6 (40)	5 (33.3)	0 (0)	2 (13.3)	0 (0)	0 (0)
Overall	48 (58.6)	18 (22.0)	1 (1.2)	7 (8.5)	2 (2.4)	3 (3.7)

#### Unadjusted Analyses

**Figure 1:**
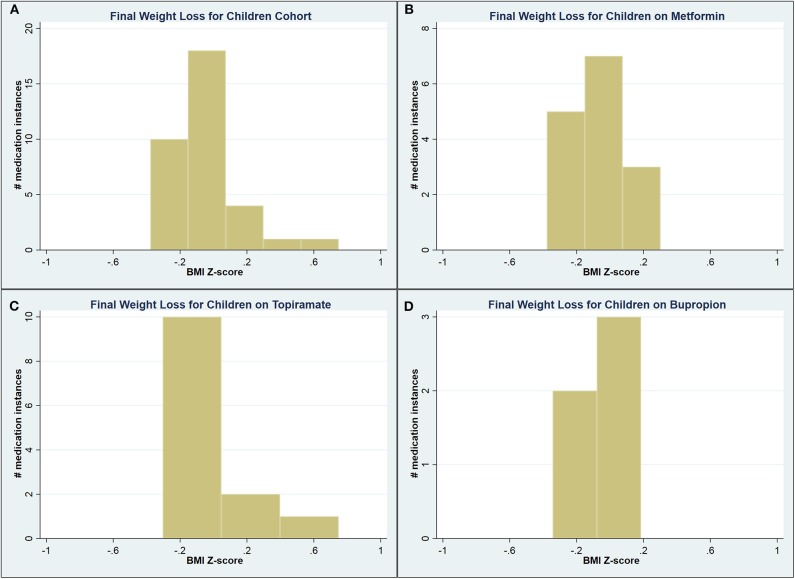
Graphical representation of the final weight loss distribution in children cohort **(A)** across all medications, **(B)** on metformin, **(C)** on topiramate, and **(D)** on bupropion.

A total of 90 medication instances were identified among children, but complete weight data (starting, nadir, and end weights) were available for only 41 (45.6%) of them. Compared to the mean starting BMIz of 2.42, children on bupropion had a lower starting BMIz of 2.21 (*p* = 0.03). No other statistically significant differences were identified on unadjusted analysis among this small cohort (Table 5). See Figure 1 for a graphic representation of the final WL distribution in this cohort.

**Table 5:** Medication characteristics and effect on weight loss, all patients - unadjusted analyses.

**Medication**	**Medication Instances, *n* (%)**	**Instances with complete weight information, *n* (%)^**¥**^**	**Pre-medication BMI, kg/m^2^ mean (95% CI)**	**Duration of use, years mean (95% CI)**	**Maximum weight loss, %TBW mean (95% CI)^**‡**^**	**Final weight loss, %TBW mean** **(95% CI)**^**‡**^
**Young adults (19–25 years old)**						
Metformin	489 (32.7)	239 (48.9)	^*^42.1 (41.2–43.1)	^*^2.50 (2.0–3.0)	3.42 (2.6–4.3)	−0.39 (−1.5–0.7)
Topiramate	378 (25.3)	166 (43.9)	^*^38.0 (37.0–39.0)	2.18 (1.9–2.4)	3.98 (2.9–5.0)	0.34 (−1.1–1.7)
Bupropion	370 (24.7)	146 (39.5)	^*^37.5 (36.4–38.7)	2.24 (2.0–2.5)	2.44 (1.0–3.8)	^*^-2.88 (−4.4–1.3)
Phentermine	142 (9.5)	75 (52.8)	^*^41.5 (39.7–43.3)	0.84 (−0.9–2.5)	4.42 (3.1–5.8)	^*^1.17 (−0.3–2.6)
Zonisamide	32 (2.1)	7 (21.9)	39.1 (31.9–46.2)	1.93 (1.2–2.7)	5.03 (−1.8–11.8)	−4.87 (−10.9–1.1)
Naltrexone	39 (2.6)	13 (33.3)	^*^32.8 (29.6–36.0)	^*^1.02 (0.5–1.5)	3.13 (0.8–5.5)	0.76 (−2.1–3.6)
Orlistat	23 (1.5)	13 (56.5)	40.9 (37.7–44.2)	^*^0.76 (0.4–1.1)	^*^0.90 (−0.5–2.3)	−2.24 (−4.8–0.3)
Liraglutide	23 (1.5)	6 (26.1)	44.7 (38.7–50.7)	^*^1.03 (0.5–1.5)	3.52 (2.1–5.0)	^*^0.69 (−0.5–1.9)
**Overall**	1,496 (100.0)	665 (44.5)	39.8 (39.3–40.4)	2.10 (1.8–2.3)	3.42 (2.9–4.0)	−0.63 (−1.3–0.0)
**Adolescents (13–18 years old)**						
Metformin	283 (45.4)	159 (56.2)	^*^2.42 (2.4–2.5)	^*^2.55 (2.3–2.8)	^*^0.07 (0.0–0.1)	−0.01 (0.0–0.0)
Topiramate	156 (25.0)	66 (42.3)	^*^2.19 (2.1–2.3)	2.22 (1.9–2.6)	0.14 (0.1–0.2)	^*^-0.08 (−0.1–0.0)
Bupropion	123 (19.7)	45 (36.6)	^*^2.15 (2.0–2.3)	2.09 (1.7–2.5)	0.09 (0.0–0.2)	−0.04 (−0.2–0.1)
Phentermine	47 (7.5)	23 (48.9)	2.36 (2.2–2.5)	^*^1.82 (1.3–2.3)	^*^0.24 (0.1–0.4)	^*^0.12 (0.0–0.2)
Zonisamide	15 (2.4)	6 (40.0)	2.15 (1.9–2.4)	1.74 (0.2–3.3)	^*^-0.08 (−0.3–0.1)	^*^-0.08 (−0.1–0.1)
**Overall**	624 (100.0)	299 (47.9)	2.32 (2.3–2.4)	2.30 (2.1–2.5)	0.09 (0.1–0.1)	−0.02 (0.0–0.0)
**Children (5–12 years old)**						
Metformin	41 (45.6)	17 (41.5)	2.50 (2.4–2.6)	2.75 (2.0–3.5)	0.03 (0.0–0.1)	−0.06 (−0.2–0.0)
Topiramate	33 (36.7)	17 (51.5)	2.39 (2.3–2.5)	2.64 (1.8–3.5)	0.11 (0.0–0.2)	0.01 (−0.1–0.2)
Bupropion	16 (17.8)	7 (43.8)	^*^2.21 (2.0–2.4)	2.70 (1.2–4.2)	0.08 (0.0–0.1)	−0.07 (−0.3–0.1)
**Overall**	90 (100.0)	41 (45.6)	2.42 (2.3–2.5)	2.70 (2.2–3.2)	0.06 (0.0–0.1)	−0.04 (−0.1–0.0)

* Statistically significant difference when compared to the overall frequency-weighted mean, p < 0.05.

¥ *Complete weight information indicates patients with both start and end weights recorded in chart. ^‡^Negative weight loss indicates weight gain*.

#### Adjusted Analyses

Multivariable analyses demonstrated higher obesity class was significantly associated with greater maximum WL (ΔBMIz 0.17 for both Class II and III, *p* = 0.002 for both; Table 6). Black children were more likely to experience a final weight gain (ΔBMIz −0.27; *p* = 0.05) when compared to whites. Similarly, compared to patients with no obesity-related illnesses, children with one or more conditions were significantly more likely to experience a final weight gain (ΔBMIz −0.78 to −0.4, *p* < 0.03; Table 7).

**Table 6:** Effect on maximum weight loss, all patients—adjusted analyses.

	**Young Adults (19–25 years old)**		**Adolescents (13–18 years old)**		**Children (5–12 years old)**	
	^**‡**^***N*** **=** **665 medication instances**		^**‡**^***N*** **=** **299 medication instances**		^**‡**^***N*** **=** **41 medication instances**	
	**%TBW (95% CI)**	***P-*value**	**ΔBMIz (95% CI)**	***P-*value**	**ΔBMIz (95% CI)**	***P-*value**
Medication^*^						
Metformin	−0.49 (−1.2–0.2)	>0.1	−0.02 (0.0–0.0)	0.06	−0.03 (−0.1–0.0)	>0.1
Topiramate	0.75 (−0.2–1.7)	>0.1	0.04 (0.0–0.1)	>0.1	0.03 (−0.1–0.1)	>0.1
Bupropion	−0.38 (−1.5–0.8)	>0.1	0.00 (−0.1–0.1)	>0.1	0.07 (−0.1–0.2)	>0.1
Phentermine	1.14 (−0.2–2.5)	>0.1	0.13 (0.0–0.3)	0.06	—	—
Zonisamide	1.15 (−5.8–8.1)	>0.1	−0.20 (−0.4–0.0)	***0.05***	—	—
Naltrexone	0.91 (−1.8–3.6)	>0.1	—	—	—	—
Orlistat	−2.83 (−4.2–1.5)	*** < 0.001***	—	—	—	—
Liraglutide	−0.93 (−3.0–1.1)	>0.1	—	—	—	—
Obesity class						
Overweight	ref	—	ref	—	ref	—
Class I (mild)	1.51 (−0.5–3.6)	>0.1	0.08 (−0.1–0.3)	>0.1	0.11 (0.0–0.3)	>0.1
Class II (moderate)	2.47 (0.4–4.6)	***0.02***	0.09 (−0.1–0.3)	>0.1	0.17 (0.1–0.3)	***0.002***
Class III (severe)	2.59 (0.7–4.5)	***0.009***	−0.01 (−0.2–0.2)	>0.1	0.17 (0.1–0.3)	***0.002***
Age at med initiation, years	0.08 (−0.2–0.4)	>0.1	0.01 (0.0–0.0)	>0.1	0.00 (0.0–0.0)	>0.1
Female sex	0.01 (−1.4–1.4)	>0.1	−0.03 (−0.1–0.0)	>0.1	−0.06 (−0.2–0.0)	>0.1
Race						
White	ref	—	ref	—	ref	—
Black	0.08 (−1.6–1.7)	>0.1	0.03 (−0.1–0.1)	>0.1	0.02 (−0.2–0.2)	>0.1
Hispanic	−0.13 (−1.6–1.4)	>0.1	−0.02 (−0.1–0.0)	>0.1	0.00 (−0.1–0.1)	>0.1
Asian	2.00 (−1.2–5.2)	>0.1	0.09 (−0.1–0.3)	>0.1	0.01 (−0.1–0.1)	>0.1
Insurance						
Private	ref	—	ref	—	ref	—
Medicaid	0.17 (−1.0–1.3)	>0.1	−0.02 (−0.1–0.0)	>0.1	−0.01 (−0.2–0.1)	>0.1
Self–pay	0.41 (−3.0–3.8)	>0.1	0.15 (0.0–0.3)	>0.1	—	—
Obesity-related illnesses, *N*						
0	ref	—	ref	—	ref	—
1-2	0.75 (−1.0–2.5)	>0.1	−0.01 (−0.1–0.1)	>0.1	−0.39 (−1.0–0.2)	>0.1
3–4	1.77 (0.1–3.5)	***0.04***	0.01 (−0.1–0.1)	>0.1	−0.42 (−1.0–0.1)	>0.1
5–6	1.92 (0.1–3.7)	***0.04***	0.04 (−0.1–0.2)	>0.1	−0.40 (−1.0–0.2)	>0.1
≥6	2.23 (−0.3–4.8)	0.09	0.12 (−0.1–0.3)	>0.1	−0.44 (−1.0–0.1)	>0.1

* Coefficient indicates difference between respective medication and the overall frequency-weighted mean.

‡ *N, patients with both pre-medication and post-medication weights recorded in chart; ref, reference group. Bold and italic value represents means-statistically significant*.

**Table 7:** Effect on Final Weight Loss, All Patients - Adjusted Analyses.

	**Young adults (19–25 years old)**		**Adolescents (13–18 years old)**		**Children (5–12 years old)**	
	^****‡****^***N*** **=** **665 medication instances**		^****‡****^***N*** **=** **299 medication instances**		^****‡****^***N*** **=** **41 medication instances**	
	**%TBW (95% CI)**	***P-*value**	**ΔBMIz (95% CI)**	***P-*value**	**ΔBMIz (95% CI)**	***P*-value**
Medication^*^						
Metformin	−0.66 (−1.5–0.2)	>0.1	0.00 (0.0–0.0)	>0.1	−0.02 (−0.1–0.0)	>0.1
Topiramate	1.47 (0.3–2.6)	***0.01***	−0.07 (−0.1–0.0)	***0.02***	−0.03 (−0.1–0.1)	>0.1
Bupropion	−1.33 (−2.7–0.0)	***0.05***	0.03 (−0.1–0.1)	>0.1	0.15 (−0.1–0.4)	>0.1
Phentermine	1.54 (0.0–3.0)	***0.05***	0.12 (0.0–0.2)	***0.003***	—	—
Zonisamide	−4.27 (−10.2–1.6)	>0.1	−0.07 (−0.2–0.0)	>0.1	—	—
Naltrexone	2.48 (−0.3–5.3)	0.08	—	—	—	—
Orlistat	−1.35 (−3.6–0.9)	>0.1	—	—	—	—
Liraglutide	−0.60 (−2.3–1.1)	>0.1	—	—	—	—
Obesity class						
Overweight	ref	—	ref	—	ref	—
Class I (mild)	3.51 (0.8–6.2)	***0.01***	0.35 (0.1–0.6)	***0.003***	—	—
Class II (moderate)	6.57 (4.0–9.1)	***<0.001***	0.33 (0.1–0.6)	***0.003***	−0.06 (−0.3–0.2)	>0.1
Class III (severe)	6.26 (3.9–8.6)	***<0.001***	0.31 (0.1–0.5)	***0.005***	0.06 (−0.1–0.2)	>0.1
Age at med initiation, years	−0.10 (−0.4–0.2)	>0.1	0.01 (0.0–0.0)	>0.1	−0.03 (−0.1–0.0)	>0.1
Female sex	−0.60 (−2.2–1.0)	>0.1	−0.01 (−0.1–0.1)	>0.1	0.03 (−0.1–0.2)	>0.1
Race						
White	ref	—	ref	—	ref	—
Black	−1.87 (−3.9–0.2)	0.07	0.04 (−0.1–0.1)	>0.1	−0.27 (−0.5–0.0)	***0.05***
Hispanic	0.25 (−1.6–2.1)	>0.1	0.00 (−0.1–0.1)	>0.1	−0.16 (−0.4–0.1)	>0.1
Asian	0.23 (−4.0–4.5)	>0.1	0.03 (−0.1–0.1)	>0.1	—	—
Insurance						
Private	ref	—	ref	—	ref	—
Medicaid	0.54 (−0.8–1.9)	>0.1	−0.01 (−0.1–0.0)	>0.1	0.07 (−0.1–0.2)	>0.1
Self-pay	−0.68 (−6.3–5.0)	>0.1	0.16 (−0.1–0.4)	>0.1	—	—
Obesity-related illnesses, *N*						
0	ref	—	ref	—	ref	—
1–2	1.31 (−2.9–5.6)	>0.1	−0.04 (−0.2–0.1)	>0.1	−0.40 (−0.7–0.1)	***0.01***
3–4	2.77 (−1.5–7.0)	>0.1	0.01 (−0.1–0.1)	>0.1	−0.46 (−0.7–0.2)	*** <0.001***
5–6	1.54 (−2.7–5.8)	>0.1	−0.07 (−0.2–0.1)	>0.1	−0.78 (−1.1–0.5)	*** <0.001***
≥6	2.02 (−3.0–7.0)	>0.1	−0.04 (−0.2–0.1)	>0.1	−0.40 (−0.7–0.1)	***0.03***

* Coefficient indicates difference between respective medication and the overall frequency-weighted mean.

‡ *N, patients with both pre-medication and post-medication weights recorded in chart. ref, reference group. Bold and italic value represents means-statistically significant*.

#### Off-Label Analyses

After removing all on-label medication instances, only metformin and topiramate had 5 or more instances with complete weight data. No statistically significant findings were identified on unadjusted analysis (Table 8), or on multivariable analysis related to maximum WL (Table 9). The previously observed relationship between black race and obesity-related illnesses and final WL remained (Table 10).

**Table 8:** Medication characteristics and effect on weight loss, off-label patients—unadjusted analyses.

**Medication**	**Medication Instances, *n* (%)**	**Instances with complete weight information, n (%)^**¥**^**	**Pre-medication BMI, kg/m^2^ mean (95% CI)**	**Duration of use, years mean (95% CI)**	**Maximum weight loss, %TBW mean (95% CI)^**‡**^**	**Final weight loss, %TBW mean** **(95% CI)**^**‡**^
**Young adults (19–25 years old)**						
Metformin	257 (33.1)	126 (49.0)	^*^41.5 (40.2–42.7)	1.73 (0.8–2.7)	2.66 (1.5–3.9)	−1.11 (−2.7–0.4)
Topiramate	299 (38.5)	142 (47.5)	^*^38.3 (37.2–39.4)	1.94 (1.7–2.2)	3.53 (2.4–4.6)	0.62 (−0.7–1.9)
Bupropion	21 (2.7)	8 (38.1)	37.8 (32.0–43.7)	1.63 (0.7–2.6)	^*^-4.91 (−9.9–0.1)	−6.68 (−14.0–0.6)
Phentermine	142 (18.3)	75 (52.8)	41.5 (39.7–43.3)	0.84 (−0.9–2.5)	4.42 (3.1–5.8)	1.17 (−0.3–2.6)
Zonisamide	16 (2.1)	6 (37.5)	40.9 (31.5–50.2)	1.23 (0.3–2.2)	3.93 (−2.3–10.2)	−2.89 (−8.3–2.5)
Naltrexone	18 (2.3)	6 (33.3)	37.8 (32.5–43.1)	1.15 (0.2–2.1)	3.75 (0.1–7.4)	^*^3.29 (1.4–5.2)
Orlistat	23 (3.0)	13 (56.5)	40.9 (37.7–44.2)	^*^0.76 (0.4–1.1)	^*^0.90 (−0.5–2.3)	−2.24 (−4.8–0.3)
**Overall**	776 (100.0)	376 (48.5)	40.2 (39.4–40.9)	1.59 (1.1–2.0)	3.19 (2.5–3.9)	−0.12 (−0.9–0.7)
**Adolescents (13–18 years old)**						
Metformin	175 (50.9)	93 (53.1)	^*^2.39 (2.3–2.5)	1.90 (1.6–2.2)	^*^0.03 (0.0–0.1)	−0.03 (−0.1–0.0)
Topiramate	122 (35.5)	52 (42.6)	^*^2.17 (2.1–2.3)	1.98 (1.6–2.4)	0.08 (0.0–0.1)	−0.05 (−0.1–0.0)
Phentermine	47 (13.7)	23 (48.9)	2.36 (2.2–2.5)	1.82 (1.3–2.3)	^*^0.24 (0.1–0.4)	^*^0.12 (0.0–0.2)
**Overall**	344 (100.0)	168 (48.8)	2.32 (2.3–2.4)	1.92 (1.7–2.1)	0.07 (0.0–0.1)	−0.01 (0.0–0.0)
**Children (5–12 years old)**						
Metformin	24 (49.0)	13 (54.2)	2.51 (2.4–2.7)	2.30 (1.4–3.2)	0.07 (0.0–0.2)	−0.03 (−0.1–0.1)
Topiramate	25 (51.0)	14 (56.0)	2.36 (2.2–2.5)	2.20 (1.4–3.0)	0.09 (0.0–0.2)	0.02 (−0.1–0.2)
**Overall**	49 (100.0)	27 (55.1)	2.43 (2.3–2.5)	2.25 (1.7–2.8)	0.08 (0.0–0.2)	0.00 (−0.1–0.1)

* Statistically significant difference when compared to the overall frequency-weighted mean, p < 0.05.

¥ Complete weight information indicates patients with both start and end weights recorded in chart.

‡ *Negative weight loss indicates weight gain*.

**Table 9:** Effect on maximum weight loss, off-label patients—adjusted analyses.

	**Young adults (19–25 years old)**		**Adolescents (13–18 years old)**		**Children (5–12 years old)**	
	^**‡**^***N*** **=** **376 medication instances**		^**‡**^***N*** **=** **168 medication instances**		^**‡**^***N*** **=** **27 medication instances**	
	**%TBW (95% CI)**	***P-*value**	**ΔBMIz (95% CI)**	***P-*value**	**ΔBMIz (95% CI)**	***P-*value**
Medication^*^						
Metformin	−0.90 (−1.7–0.1)	***0.04***	−0.04 (−0.1–0.0)	***0.006***	0.00 (−0.1–0.1)	>0.1
Topiramate	0.57 (−0.3–1.4)	>0.1	0.02 (0.0–0.1)	>0.1	0.00 (−0.1–0.1)	>0.1
Bupropion	−9.15 (−14.6–3.6)	***0.001***	—	—	—	—
Phentermine	1.56 (0.2–2.9)	***0.02***	0.15 (0.0–0.3)	***0.03***	—	—
Zonisamide	0.07 (−6.9–7.1)	>0.1	—	—	—	—
Naltrexone	1.80 (−3.9–7.5)	>0.1	—	—	—	—
Orlistat	−2.06 (−3.7–0.4)	***0.01***	—	—	—	—
Obesity class						
Overweight	ref	—	ref	—	ref	—
Class I (mild)	2.41 (−0.2–5.0)	0.07	0.18 (0.0–0.3)	***0.02***	—	—
Class II (moderate)	4.33 (1.9–6.7)	***<0.001***	0.18 (0.0–0.3)	***0.03***	0.04 (−0.2–0.3)	>0.1
Class III (severe)	3.04 (0.7–5.4)	***0.01***	0.14 (0.0–0.3)	0.07	0.11 (−0.1–0.3)	>0.1
Age at med initiation, years	0.33 (0.0–0.7)	0.07	0.00 (0.0–0.0)	>0.1	−0.01 (−0.1–0.1)	>0.1
Female sex	−0.75 (−2.9–1.4)	>0.1	−0.04 (−0.1–0.0)	>0.1	−0.14 (−0.3–0.0)	>0.1
Race						
White	ref	—	ref	—	ref	—
Black	−0.59 (−2.6–1.4)	>0.1	0.01 (−0.1–0.1)	>0.1	—	—
Hispanic	−1.75 (−3.6–0.1)	0.06	−0.06 (−0.1–0.0)	***0.03***	−0.18 (−0.4–0.1)	>0.1
Asian	−2.78 (−7.3–1.8)	>0.1	−0.02 (−0.1–0.1)	>0.1	0.01 (−0.2–0.2)	>0.1
Insurance						
Private	ref	—	ref	—	ref	—
Medicaid	−0.30 (−1.8–1.2)	>0.1	−0.01 (−0.1–0.0)	>0.1	0.12 (−0.1–0.3)	>0.1
Self-pay	0.02 (−3.5–3.5)	>0.1	0.04 (−0.1–0.2)	>0.1	—	—
Obesity-related illnesses, *N*						
0	ref	—	ref	—	ref	—
1–2	0.18 (−1.8–2.2)	>0.1	−0.02 (−0.1–0.1)	>0.1	−0.19 (−0.7–0.3)	>0.1
3–4	−0.05 (−2.0–2.0)	>0.1	−0.04 (−0.1–0.1)	>0.1	−0.40 (−0.9–0.1)	0.08
5–6	0.07 (−2.3–2.4)	>0.1	−0.06 (−0.2–0.1)	>0.1	—	—
≥6	0.98 (−3.5–5.5)	>0.1	−0.13 (−0.3–0.1)	>0.1	−0.46 (−1.1–0.2)	>0.1

* Coefficient indicates difference between respective medication and the overall frequency-weighted mean.

‡ *N, patients with both pre-medication and post-medication weights recorded in chart. ref, reference group. Bold and italic value represents means-statistically significant*.

**Table 10:** Effect on final weight loss, off-label patients—adjusted analyses.

	**Young Adults (19–25 years old)**		**Adolescents (13–18 years old)**		**Children (5–12 years old)**	
	^****‡****^***N*** **=** **376 medication instances**		^****‡****^***N*** **=** **168 medication instances**		^****‡****^***N*** **=** **27 medication instances**	
	**%TBW (95% CI)**	***P-*value**	**ΔBMIz (95% CI)**	***P-*value**	**ΔBMIz (95% CI)**	***P-*value**
Medication^*^						
Metformin	−1.68 (−2.8–0.6)	***0.003***	−0.02 (0.0–0.0)	***0.03***	0.01 (−0.1–0.1)	>0.1
Topiramate	1.31 (0.3–2.3)	***0.01***	−0.03 (−0.1–0.0)	>0.1	−0.01 (−0.1–0.1)	>0.1
Bupropion	−6.79 (−13.2–0.4)	***0.04***	—	—	—	—
Phentermine	1.37 (0.0–2.8)	0.06	0.15 (0.1–0.2)	***<0.001***	—	—
Zonisamide	−2.37 (−7.7–3.0)	>0.1	—	—	—	—
Naltrexone	2.39 (0.3–4.4)	***0.02***	—	—	—	—
Orlistat	−1.77 (−4.2–0.6)	>0.1	—	—	—	—
Obesity class						
Overweight	ref	—	ref	—	ref	—
Class I (mild)	5.86 (2.0–9.8)	***0.003***	0.07 (−0.1–0.3)	>0.1	—	—
Class II (moderate)	8.45 (4.8–12.1)	***<0.001***	0.10 (−0.1–0.3)	>0.1	−0.05 (−0.3–0.2)	>0.1
Class III (severe)	7.74 (4.1–11.3)	***<0.001***	0.08 (−0.1–0.3)	>0.1	0.01 (−0.2–0.2)	>0.1
Age at med initiation, years	0.18 (−0.2–0.6)	>0.1	0.01 (0.0–0.0)	>0.1	−0.02 (−0.1–0.0)	>0.1
Female sex	−0.83 (−3.0–1.3)	>0.1	−0.01 (−0.1–0.0)	>0.1	−0.02 (−0.3–0.3)	>0.1
Race						
White	ref	—	ref	—	ref	—
Black	−2.64 (−4.9–0.4)	***0.02***	0.09 (0.0–0.2)	***0.005***	−0.28 (−0.5–0.1)	***0.01***
Hispanic	−1.53 (−3.6–0.6)	>0.1	0.04 (0.0–0.1)	>0.1	−0.11 (−0.4–0.2)	>0.1
Asian	−1.62 (−7.8–4.5)	>0.1	0.05 (0.0–0.1)	>0.1	—	—
Insurance						
Private	ref	—	ref	—	ref	—
Medicaid	0.51 (−1.1–2.1)	>0.1	0.03 (0.0–0.1)	>0.1	0.08 (−0.1–0.3)	>0.1
Self-pay	−1.72 (−10.4–7.0)	>0.1	−0.03 (−0.1–0.0)	>0.1	—	—
Obesity-related illnesses, *N*						
0	ref	—	ref	—	ref	—
1–2	−0.97 (−4.5–2.5)	>0.1	0.01 (−0.1–0.1)	>0.1	−0.32 (−0.8–0.2)	>0.1
3–4	0.42 (−2.9–3.7)	>0.1	−0.01 (−0.1–0.1)	>0.1	−0.40 (−0.7–0.1)	***0.02***
5–6	−1.36 (−4.8–2.1)	>0.1	−0.10 (−0.2–0.0)	***0.05***	—	—
≥6	1.51 (−3.3–6.3)	>0.1	−0.22 (−0.6–0.2)	>0.1	−0.39 (−0.8–0.0)	0.06

* Coefficient indicates difference between respective medication and the overall frequency-weighted mean.

‡ *N, patients with both pre-medication and post-medication weights recorded in chart. ref, reference group. Bold and italic value represents means-statistically significant*.

### Adolescents

Metformin was again the most commonly prescribed medication among adolescents (280; 56.9%), even after excluding patients with potential on-label indications (173; 57.9%; Table 3). Most adolescents continued to take the study medication at the time of data extraction (45.5%), while 31.7% had discontinued it for unknown/unspecified reasons. See Table 4 for the most common reasons for medication discontinuation.

#### Unadjusted Analyses

A total of 624 medication instances were identified among adolescent patients, 299 (47.9%) of which had complete weight data recorded in the chart (Table 5). Compared to the mean starting BMIz of 2.32, adolescents on metformin had a higher starting BMIz (2.42; *p* < 0.001), while it was lower for patients on topiramate (BMIz 2.19; *p* = 0.002) and bupropion (BMIz 2.15, *p* = 0.004).

**Figure 2:**
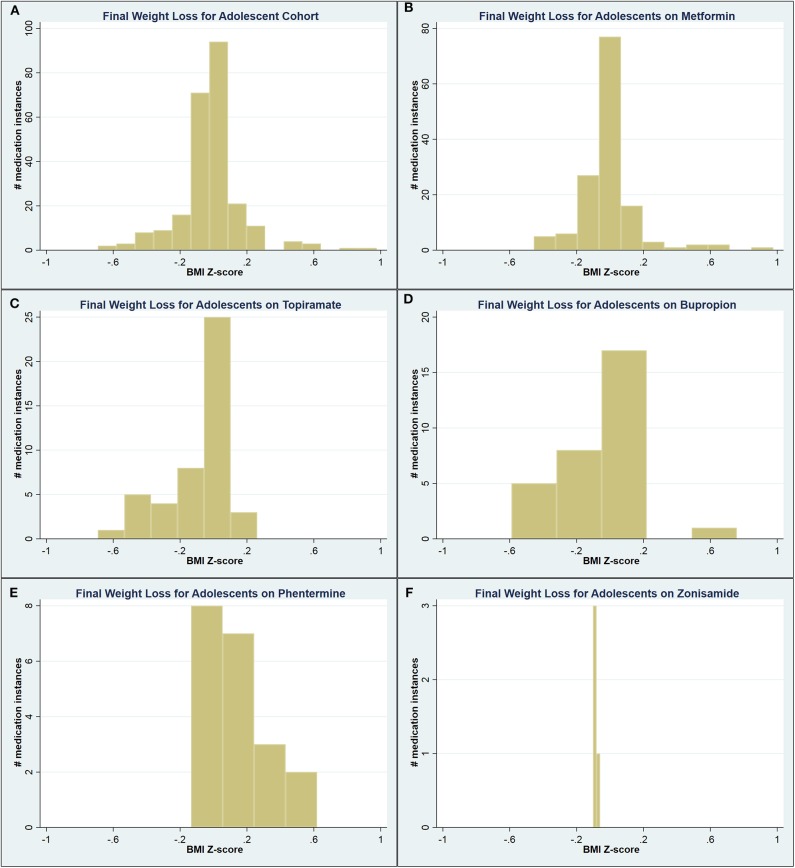
Graphical representation of the final weight loss distribution in adolescent cohort **(A)** across all medications, **(B)** on metformin, **(C)** on topiramate, **(D)** on bupropion, **(E)** on phentermine, and **(F)** on zonisamide.

Compared to the respective means, adolescents on phentermine experienced a greater maximum WL (0.24 vs. 0.09 ΔBMIz; *p* = 0.003) and final WL (0.12 vs. −0.02 ΔBMIz; *p* = 0.001), while patients on topiramate and zonisamide experienced a significantly lesser final WL (−0.08 ΔBMIz for both; *p* = 0.02 and < 0.001, respectively). A significant proportion of adolescents ultimately experienced weight gain by the time of medication discontinuation (Figure 2).

#### Adjusted Analyses

Multivariable analyses demonstrated adolescents on phentermine experienced a final WL 0.12 ΔBMIz greater than the mean (*p* = 0.003), while those on topiramate experienced a final WL 0.07 ΔBMIz lesser than the mean (*p* = 0.02). Higher weight class at the time of medication initiation was associated with greater final WL (Table 7).

#### Off-Label Analyses

After removing all on-label indications, metformin, topiramate, and phentermine remained. Unadjusted analyses were largely unchanged from prior (Table 8). Multivariable analyses demonstrated patients on phentermine experienced a 0.15 ΔBMIz greater maximum WL and final WL (*p* = 0.003 and < 0.001, respectively) when compared to the respective means, while patients on metformin experienced a 0.04 (*p* = 0.006) and 0.02 (*p* = 0.03) ΔBMIz lesser maximum WL and final WL respectively (Tables 9, 10).

### Young Adults

In line with the other two cohorts, metformin was the most commonly prescribed medication among young adults overall (484; 42.3%), but topiramate was the most commonly prescribed off-label medication (298; 46.3%, Table 3). Similar to children and adolescents, a large number of young adults continued to take the medication at the time of data extraction (36.8%), while a slightly higher proportion had discontinued it for unknown/unspecified reasons (37.3%; Table 4).

#### Unadjusted Analyses

**Figure 3:**
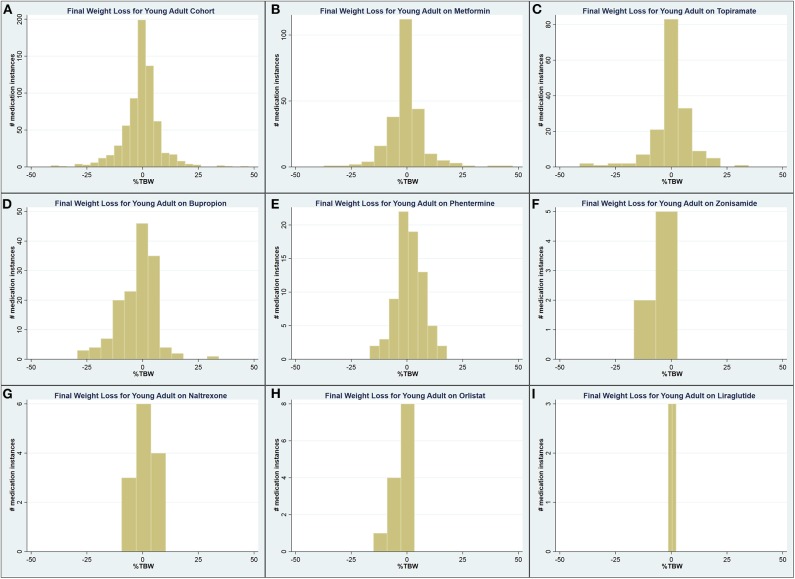
Graphical representation of the final weight loss distribution in young adult cohort **(A)** across all medications, **(B)** on metformin, **(C)** on topiramate, **(D)** on bupropion, **(E)** on phentermine, **(F)** on zonisamide, **(G)** on naltrexone, **(H)** on orlistat, and **(I)** on liraglutide.

A total of 1,496 medication instances among young adult patients were identified, 665 (44.5%) of which had complete weight data recorded in the chart. Among these, patients on metformin and phentermine had a higher starting BMI compared to the mean, while this was lower for patients on topiramate, bupropion, and naltrexone (Table 5). Patients on orlistat experienced a lesser maximum WL compared to the mean (0.90 vs. 3.42 %TBW; *p* = 0.001), while patients on phentermine experienced a greater final WL (1.17 vs. −0.63 %TBW; *p* = 0.02). Figure 3 contains the overall and medication-specific distribution of final WL among young adults.

#### Adjusted Analyses

Young adult patients on orlistat experienced a 2.83%TBW lesser maximum WL when compared to themean (*p* < 0.001), while patients on phentermine and topiramate experienced a 1.54 (*p* = 0.05) and 1.47 (*p* = 0.01) %TBW greater final WL compared to the mean, respectively (Tables 6, 7). Similar to the findings among adolescents, higher weight class at medication initiation was associated with higher final WL.

#### Off-Label Analyses

After removing all patients with on-label indications, results were similar to prior. Patients on metformin had a higher starting BMI compared to the mean (41.5 vs. 40.2 kg/m^2^; *p* = 0.005), while it was lower for patients on topiramate (38.3 kg/m^2^, *p* < 0.001; Table 8). Both patients on Orlistat (0.9%TBW; *p* = 0.003) and Bupropion (−4.91 %TBW; *p* = 0.001) had significantly lesser maximum WL compared to the mean (Table 9). The only medication with a final WL significantly different than the mean (−0.12%TBW) was naltrexone (3.29%TBW; *p* < 0.001). Patients on phentermine trended toward a greater final WL (1.17 %TBW) than the mean but this relationship did not reach statistical significance (*p* = 0.06; Table 10).

## Discussion

Our study describes the current prescribing patterns of weight-modifying pharmacotherapy amongst children, adolescents and young adults in a large unified health care system. This study also distinguishes between overall and off-label prescribing to assess the effectiveness of these medications in achieving weight modification, regardless of the specific indication. Due to sampling size, the strongest conclusions may be drawn from the adolescent and young adult cohorts. In these patient populations, phentermine appears to most consistently support WL, which is statistically significant in our study, though clinically small. Interpretation of phentermine's true weight modification benefits, as well as those effects of the other medications, however, are likely minimized based on their comparison to the mean rather than a control group not taking weight-modifying pharmacotherapy. Generally, higher obesity classification at medication initiation was associated with greater final WL. The analysis also reveals that metformin was overall the most commonly prescribed medication for both on- and off-label use among children and adolescents; however, in the adjusted analyses, it was associated with statistically significant negative final WL (i.e., weight gain) among adolescents and young adults for whom it was prescribed off-label compared to the mean. To our knowledge, this study is the first of its kind to explore this breadth of medication use with a focus on WL across a spectrum of ages from 5 to 25 years old.

The retrospective nature of our study gives rise to some important limitations. Though start and end dates of medications were manually verified by a team of clinicians, chart review is only as good as the quality of the data documented, which means that medication duration should be interpreted with caution. Additionally, the lack of a control group and our inability to account for confounding lifestyle modifications, such as diet quality and physical activity, make it difficult to fully interpret the absolute magnitude of effectiveness of the medications studied as mentioned above. Several other limitations are worth noting. First, given that we chose to limit our assessment of a medication's effect on WL to its exclusive dates of administration, we are unable to comment on the synergistic effect that multiple medications taken together may have. Second, our study did not capture medication dosing, which could play an important role and should be investigated further in future studies (35). Despite significant effort to determine reasons for medication discontinuation as part of the manual chart review, it is notable that this reason was unspecified for approximately one-third of patients. Lastly, this study did not account for confounders such as the simultaneous use of other medications associated with weight gain (18), which could be of particular importance given the high rates of anxiety (62.3%) and depression (55.7%) noted.

Despite these limitations, our study is the first to our knowledge to evaluate prescribing patterns and usage of a variety of medications among children, adolescents, and young adults, in the context of overweight and obesity. Strengths of this study include its large sample size based out of a multicenter academic health system with an established tertiary care facility to treat pediatric obesity. Providers in this center include a multidisciplinary team (i.e., physician, dietitian, psychologist) trained specifically in the management of both pediatric and adult obesity and therefore are more inclined and familiar with prescribing these medications for the indication of WL.

Obesity is a heterogeneous, multifactorial disease that is best treated with multimodal therapies, including lifestyle modification, pharmacotherapy, and surgery as an integrated continuum of care (36, 37). No studies have demonstrated a durable, sustained WL through lifestyle modifications alone (15).

A systematic review by O'Connor and colleagues found that children and adolescents following a traditional lifestyle modification plan require a minimum of 26 h over a 6–12-months period of contact with providers to achieve an average 1.0 kg/m^2^ BMI reduction (38). Another study which evaluated age and obesity class, showed that behavioral interventions are less effective in terms of BMI reduction among adolescents (14–16 years old) when compared to children (6–9 years) (39). For adolescents who have experienced insufficient weight control with lifestyle modification therapy and who do not meet criteria for MBS, pharmacotherapy could serve as an important treatment option (40). Our study corroborates this and suggests that phentermine could be a useful first-line pharmacologic option for adolescents 13–18 years old.

Pharmacotherapy, in particular, directly targets biologic adaptations and counterregulatory mechanisms through action on appetite and hunger (39, 41). Note, that with any of the interventions utilized to treat obesity, there is an anticipated metabolic adaptation to WL. It is not uncommon for patients with obesity to experience WL followed by weight regain (42). In a 2011 paper delving into the physiology of WL and regain, Maclean and colleagues summarize this phenomenon and conclude that treatments must therefore be wholly exhaustive and redundant to overcome this biology (43).

Though weight reduction in our study was modest, studies have shown that initial WL with behavioral changes and adjunctive pharmacotherapy is enough to reduce significant health risks associated with many obesity-related conditions (44). In a systematic review for the US Preventive Services Task Force (USPSTF) of recent randomized-controlled trials, researchers found that the control groups were more likely to continue to gain excess weight compared to the pharmacologic treatment groups. They concluded that even an arrest in weight gain may prove clinically significant over a lifetime (38).

Unfortunately, there are several barriers to medication adherence including inadequate compliance, adverse effects, insurance coverage and other reasons for medication discontinuation as demonstrated in this study. There is also prescriber resistance to AOM use while awaiting results of FDA-approved long-term outcome trials and inadequate obesity education across the continuum from medical school to fellowship (19, 45). Hence, most current prescribers of AOMs remain endocrinologists who are more comfortable with managing these medications for other indications despite availability of certification by the American Board of Obesity Medicine (ABOM). A recent study demonstrates that pediatricians are least likely to become certified by the ABOM to treat patients with obesity despite the consistent rise of obesity among the pediatric population (19, 46). Still, off-label systemic medication prescribing for unapproved conditions in pediatrics has increased in recent years (47). As seen in this study, most of the medications on market and in use for separate indications are FDA-approved for adults for obesity and are often used off-label to treat obesity in the pediatric population.

A recent policy statement from the American Academy of Pediatrics (AAP) recommended improved access for pediatric patients “to multidisciplinary programs that provide high quality pediatric metabolic and bariatric surgery” with no lower age limit given emerging long-term outcome data (15, 16, 48). With this shift in policy comes an even greater role for pharmacotherapy treatment as prior studies have found that AOMs serve as a useful adjunct in postsurgical patients following bariatric surgery to treat inadequate WL or weight regain (49–53). Unfortunately, there also remain significant barriers to MBS for the management of pediatric obesity relating to racial disparities in that white adolescents are more likely to undergo MBS despite higher rates of obesity existing amongst black and Hispanic youth (54).

Medications are uniquely suited for use in the pediatric population because children with obesity have a greater degree of disease plasticity (4). The fact remains that there are far greater FDA-approved options available to adults with obesity than for children and adolescents. This study supports the need for more clinical trials and greater efforts which focus on FDA-approval of medications for WL in pediatrics to target obesity early.

In conclusion, our study demonstrates that AOMs may play a role in weight management for adolescents and young adults with obesity and should be considered an important component of a multimodal approach to managing this severe and deleterious condition. Their use may become particularly important as patients who undergo lifestyle modification or MBS continue to face challenges with attaining and maintaining appropriate, durable WL. Prospective studies should be conducted to better understand the impact of these medications in the pediatric population.

## Data Availability Statement

All datasets generated for this study are included in the article/Supplementary Material.

## Ethics Statement

The studies involving human participants were reviewed and approved by the Partners Institutional Review Board (IRB) prior to written informed consent from the participants' legal guardian/next of kin was not required to participate in this study in accordance with the national legislation and the institutional requirements.

## Author Contributions

FS and KJC contributed to conception and design of the study. KJC and NP organized the database. KJC, SS, and KSC completed manual chart review. NP performed the statistical analysis. KSC wrote the first draft of the manuscript. NP and KSC wrote sections of the final manuscript. All authors contributed to manuscript revision, read, and approved the submitted version.

## Conflict of Interest

Employment by Novo Nordisk (SS); medical advisor to Novo Nordisk (FS). The remaining authors declare that the research was conducted in the absence of any commercial or financial relationships that could be construed as a potential conflict of interest.
