# Supplementary material for: Pharmacotherapy for the Treatment of Overweight and Obesity in Children, Adolescents, and Young Adults in a Large Health System in the US
Source: Front Endocrinol (Lausanne). 2020 May 13;11:290. doi: 10.3389/fendo.2020.00290 (PMC7237714; doi:10.3389/fendo.2020.00290)
Supplement: Supplementary file 1 [file Table_1.DOCX]

***Supplemental Table 1: Medication Effects on Weight Loss - Adjusted Analysis***

/* DM

250x Diabetes mellitus

648.0x Diabetes mellitus complicating pregnancy childbirth or the puerperium

E10 Type 1 diabetes mellitus

E11 Type 2 diabetes mellitus

O24.0x Pre-existing type 1 diabetes mellitus, in pregnancy, childbirth and the puerperium

O24.1x Pre-existing type 2 diabetes mellitus, in pregnancy, childbirth and the puerperium

O24.3x Unspecified pre-existing diabetes mellitus in pregnancy, childbirth and the puerperium

O24.8x Other pre-existing diabetes mellitus in pregnancy, childbirth, and the puerperium

/* HTN

401.x Essential hypertension

402.x Hypertensive heart disease

403.x Hypertensive chronic kidney disease

404.x Hypertensive heart and chronic kidney disease

405.x Secondary hypertension

I10.x Essential (primary) hypertension

I11.x Hypertensive heart disease

I12.x Hypertensive chronic kidney disease

I13.x Hypertensive heart and chronic kidney disease

I15.x Secondary hypertension

/* HLD

272.x Disorders of lipoid metabolism

E78.x Disorders of lipoprotein metabolism and other lipidemias

/* HLD

272.x Disorders of lipoid metabolism

E78.x Disorders of lipoprotein metabolism and other lipidemias

/* Pickwickian syndrome

278.03 Obesity hypoventilation syndrome

E66.2 Morbid (severe) obesity with alveolar hypoventilation

/* NASH

571.8 Other chronic nonalcoholic liver disease

K76.0 Fatty (change of) liver, not elsewhere classified

K76.89 Other specified diseases of liver

/* IIH

348.2 Benign intracranial hypertension

G93.2 Benign intracranial hypertension

/* GERD

530.11 Reflux esophagitis

530.81 Esophageal reflux

K21.x Gastro-esophageal reflux disease

/* Epilepsy

345x Epilepsy and recurrent seizures

780.39 Other convulsions

G40x Epilepsy and recurrent seizures

R56.9 Unspecified convulsions

/* Mental health disorders

296.2 Major depressive disorder single episode

296.3 Major depressive disorder recurrent episode

300.4 Dysthymic disorder

311 Depressive disorder, not elsewhere classified

F32 Major depressive disorder, single episode

F33 Major depressive disorder, recurrent

F34.1 Dysthymic disorder

/* Anxiety

300 Anxiety, dissociative and somatoform disorders

F40-F48 Anxiety, dissociative, stress-related, somatoform and other nonpsychotic mental disorders

/* Personality disorders

301 Personality disorders

F60 Specific personality disorders

/* ADD/ADHD

314.0 Attention deficit disorder of childhood

F90 Attention-deficit hyperactivity disorders

/* Tobacco use/dependence

305.1 Tobacco use disorder

F17 Nicotine dependence

/* Alcohol dependence/abuse

291x Alcohol-induced mental disorders

303x Alcohol dependence syndrome

305.0x Nondependent alcohol abuse

F10x Alcohol related disorders

O99.31x Alcohol use complicating pregnancy, childbirth, and the puerperium

/* Opioid dependence/abuse

304.0 Opioid type dependence

305.5 Nondependent opioid abuse

F11 Opioid related disorders
